# Remediation of Pb-contaminated soil using biochar-based slow-release P fertilizer and biomonitoring employing bioindicators

**DOI:** 10.1038/s41598-022-27043-8

**Published:** 2023-01-30

**Authors:** María Paula Acosta-Luque, Julián E. López, Nancy Henao, Daniela Zapata, Juan C. Giraldo, Juan F. Saldarriaga

**Affiliations:** 1 Department of Civil and Environmental Engineering, Universidad de los Andes, Carrera 1Este #19A-40, 111711 Bogotá, Colombia; 2 Facultad de Arquitectura e Ingeniería, Institución Universitaria Colegio Mayor de Antioquia, Carrera 78 #65–46, 050034 Medellín, Colombia; 3 Faculty of Engineering, Universidad de Medellín, Carrera 87 #30–65, 050026 Medellín, Colombia

**Keywords:** Biogeochemistry, Environmental sciences, Solid Earth sciences

## Abstract

Soil contamination by Pb can result from different anthropogenic sources such as lead-based paints, gasoline, pesticides, coal burning, mining, among others. This work aimed to evaluate the potential of P-loaded biochar (Biochar-based slow-release P fertilizer) to remediate a Pb-contaminated soil. In addition, we aim to propose a biomonitoring alternative after soil remediation. First, rice husk-derived biochar was obtained at different temperatures (450, 500, 550, and 600 °C) (raw biochars). Then, part of the resulting material was activated. Later, the raw biochars and activated biochars were immersed in a saturated KH_2_PO_4_ solution to produce P-loaded biochars. The ability of materials to immobilize Pb and increase the bioavailability of P in the soil was evaluated by an incubation test. The materials were incorporated into doses of 0.5, 1.0, and 2.0%. After 45 days, soil samples were taken to biomonitor the remediation process using two bioindicators: a phytotoxicity test and enzyme soil activity. Activated P-loaded biochar produced at 500 °C has been found to present the best conditions for soil Pb remediation. This material significantly reduced the bioavailability of Pb and increased the bioavailability of P. The phytotoxicity test and the soil enzymatic activity were significantly correlated with the decrease in bioavailable Pb but not with the increase in bioavailable P. Biomonitoring using the phytotoxicity test is a promising alternative for the evaluation of soils after remediation processes.

## Introduction

Currently, it is sought that the products used for the treatment of contaminated agricultural soils, apart from their remedial capacity, can be used as fertilizers that provide nutrients^1,2^. Thus, soil treatment alternatives can be more attractive to farmers, being able to be easily coupled to agronomic practices for crop management. In the last decade, the possible use and application of biochar from different residual biomasses has been investigated as an alternative for the removal of contaminants from the soil and improvement of its physicochemical properties^3,4^. Biochar has a significant content of plant mineral nutrients, which can promote soil fertility. It is in this context that the use of biochar can be a promising soil treatment alternative^3–9^.

Biochars alone cannot compete with fertilizer in terms of mineral nutrient contributions. Some authors have found that when biochar ages in the soil, phosphorus is transformed into low bioavailability compounds such as hydroxyapatite (Ca-bound compounds)^10^. Nevertheless, it has also been shown that, over time, these low-solubility compounds can transform into soluble forms of phosphorus^10^.

Therefore, biochar-derived fertilizers are formulated by loading nutrients into the material, thus improving their bioavailable concentration in biochars, making them more competitive from a soil fertility point of view^1,2,5,6^. In the case of phosphorus (P), which is one of the most limiting nutrients in tropical soils due to its fixation in the soil, it has been shown that P-loaded biochars are an efficient option for managing the fertilization of this nutrient. Similarly, it has been shown that biochar has a great capacity to adsorb phosphorus, which makes it a good material to develop slow-release fertilizers^11^. Sepúlveda-Cadavid et al.^12^ have found that in comparison with traditional fertilization and the co-application of biochar and traditional fertilizer, the use of a biochar derived from corn residues loaded with P improved the bioavailability of this nutrient, in addition to increasing its absorption and the nutritional quality of the spinach cultivation.

Despite their wide use in plant nutrition, these materials have not yet been deeply investigated for their use as remedial agents for polluted soils. It has recently been shown that this type of fertilizer could immobilize contaminants such as lead (Pb), due to the high concentration of P after the recharge processes of the latter^13–15^. In the meantime, studies have reported an increase in the concentrations of metals such as Pb in soils due to anthropogenic activities^16^. The increase of Pb in soils has been evidenced and is generally above the maximum allowable, generating risks to human health, especially for children^16^.

It has been proven that after P loading, biochars derived from agro-industrial residues have improved their ability to adsorb Pb from aqueous solutions, reaching adsorption values of between 36 and 162 mg/g^17^. Other studies have shown that, after phosphorus loading, biochar significantly increases its ability to immobilize lead by more than 90%^18^.

The effectiveness of these materials to immobilize P has been reported in soils by mechanisms such as co-precipitation^13–15^. However, these studies have been limited, and there have been no systematic studies to date that evaluate the various production processes of these P-loaded biochar-derived fertilizers or what the recommended production conditions are for the design of this type of material. Such studies are necessary to develop design parameters to produce engineered biochar.

Although multiple studies have been developed where the effect of traditional P fertilization on the bioavailability of Pb in soils has been evaluated, the use of these is unsustainable. On the one hand, prices can become restrictive for farmers, especially in underdeveloped countries^19–21^. Instead, the environmental impacts associated with the leaching of P lead to the possibility of exceeding planetary limits^22,23^.

The use of selected bioindicators as biomonitors facilitates the assessment of a contaminant’s bioavailability and comparison among sites^24^. A bioindicator is a biological response to chemical(s) that provides a measure of exposure and, in some cases, the toxic effect of chemical(s)^24^. During soil remediation processes, it is very important to use biomarkers that indicate how much toxicity decreases due to changes in the concentration and chemical speciation of contaminants^25,26^. In the case of P-loaded biochar, no biomonitoring in the literature evaluates both the bioavailability of Pb and P and the response of biological systems as indicators of toxicity.

The study of biochar produced at different temperatures and both activated and inactivated is important to produce biochar loaded with P and to evaluate this product in the remediation of soils contaminated with Pb. This is because it is necessary to understand the behavior of biochar in the Pb adsorption process, determining the best conditions to produce the material with the greatest potential. Consequently, in this study, biochar produced from rice husk residues has been developed, using a fixed bed reactor^27–29^. This biochar has been activated in order to check if the process improves the surface area and, therefore, the efficiency of phosphorus adsorption (P-loaded biochar). In the same way, the effect of the application of biochar loaded with phosphorus on its bioavailability and Pb remediation in both activated and inactivated biochar has been determined. Finally, this study proposes a simple alternative to biomonitoring the soil after the remediation process, using radish and enzyme activity in the soil.

## Results and discussion

### Soil pH and electrical conductivity

The values of bioavailability of lead and phosphorus, as well as pH and electrical conductivity, are presented in Tables 4 and 5. Application of biochar increases soil pH, and this increase was maintained for a period of 45 days. It is observed that the increase in the application rate generated significant increases in the soil pH. P-loaded biochar derived from biochar produced at 550 °C was the material that generated the greatest increase in soil pH. The increase in pH of soil treated with P-loaded biochar is due to the liming effect of these materials^12,8^. The liming effect of biochars has been related to the presence of basic cations, carbonates, and soluble organic compounds^30^. These results show that despite the P loading process, the P-loaded biochars did not lose their lime capacity. Like pH, EC increased in all soils treated with P-loaded biochars. Increases in EC are explained by the release of cations and anions from the materials into the soil solution since EC is an indicator of the ions present in solution^12^. This can be related to the increase in bioavailable P in the soil as well as possible ion exchange between the cations contained in the biochar (e.g., Ca^2+^ and Mg^2+^) and Pb (e.g., Pb^2+^)^12,8^. The results suggest that the use of these materials increases soil pH and ion exchange, which can have a significant effect on the bioavailability of heavy metals such as Pb^8^ (Tables 1, 2).

**Table 1:** Changes in Pb and P bioavailability and in soil pH and electrical conductivity (EC) after application of P-loaded biochar produced from raw biochar.

Treatment	Pb (mg kg^−1^)		Olsen-P (mg kg^−1^)		pH		EC (µS cm^−1^)	
	Day 15	Day 45	Day 15	Day 45	Day 15	Day 45	Day 15	Day 45
Control	19.37	14.53	591.33	626.33	6.29	6.24	154	141
0.5% Biochar 450 °C	0.66	0.66	731.07	929.91	6.29	6.35	130.4	212
1.0% Biochar 450 °C	0.66	0.66	856.10	1053.21	6.30	6.45	173.2	224
2.0% Biochar 450 °C	2.42	0.66	995.07	1049.74	6.32	6.43	203	267
0.5% Biochar 500 °C	0.66	0.66	790.98	949.01	6.32	6.34	149.1	255
1.0% Biochar 500 °C	0.66	0.66	866.52	1238.39	6.35	6.28	154.2	298
2.0% Biochar 500 °C	0.66	0.66	882.15	1020.22	6.37	6.39	174.5	347
0.5% Biochar 550 °C	7.27	0.66	1472.47	1436.53	6.48	6.41	150.5	281
1.0% Biochar 550 °C	4.84	2.42	1449.97	1371.33	6.50	6.33	169.2	328
2.0% Biochar 550 °C	4.84	2.42	1667.46	1101.84	6.60	6.41	176.4	386
0.5% Biochar 600 °C	7.27	2.42	972.42	933.38	6.46	6.43	103	317
1.0% Biochar 600 °C	7.27	2.42	1397.47	909.07	6.54	6.32	118	358
2.0% Biochar 600 °C	7.27	4.84	1524.97	1430.38	6.67	6.38	202	361

**Table 2:** Changes in Pb and P bioavailability and in soil pH and electrical conductivity (EC) after application of P-loaded biochar produced from activated biochar.

Treatment	Pb (mg kg^−1^)		Olsen-P (mg kg^−1^)		pH		EC (µS cm^−1^)	
	Day 15	Day 45	Day 15	Day 45	Day 15	Day 45	Day 15	Day 45
Control	19.37	14.53	591.33	626.33	6.29	6.24	154	141
0.5% Biochar 450 °C	0.66	0.66	3039.91	672.89	6.33	6.42	218	231
1.0% Biochar 450 °C	0.66	0.66	2832.42	792.72	6.35	6.43	280	238
2.0% Biochar 450 °C	2.42	0.66	3227.40	723.25	6.38	6.48	364	273
0.5% Biochar 500 °C	0.66	0.66	2237.44	785.77	6.32	6.35	332	236
1.0% Biochar 500 °C	0.66	0.66	2332.44	811.82	6.34	6.38	343	318
2.0% Biochar 500 °C	0.66	0.66	2647.42	922.96	6.39	6.44	356	369
0.5% Biochar 550 °C	7.27	0.66	2170.30	631.21	6.37	6.39	256	217
1.0% Biochar 550 °C	2.42	2.42	2267.44	683.31	6.45	6.41	255	222
2.0% Biochar 550 °C	7.27	2.42	2442.43	707.62	6.52	6.56	293	235
0.5% Biochar 600 °C	7.27	2.42	2567.43	653.78	6.41	6.32	287	266
1.0% Biochar 600 °C	7.27	2.42	2337.44	707.62	6.52	6.35	290	282
2.0% Biochar 600 °C	7.27	2.42	2637.43	731.93	6.63	6.38	292	292

### Soil P bioavailability

**Figure 1:**
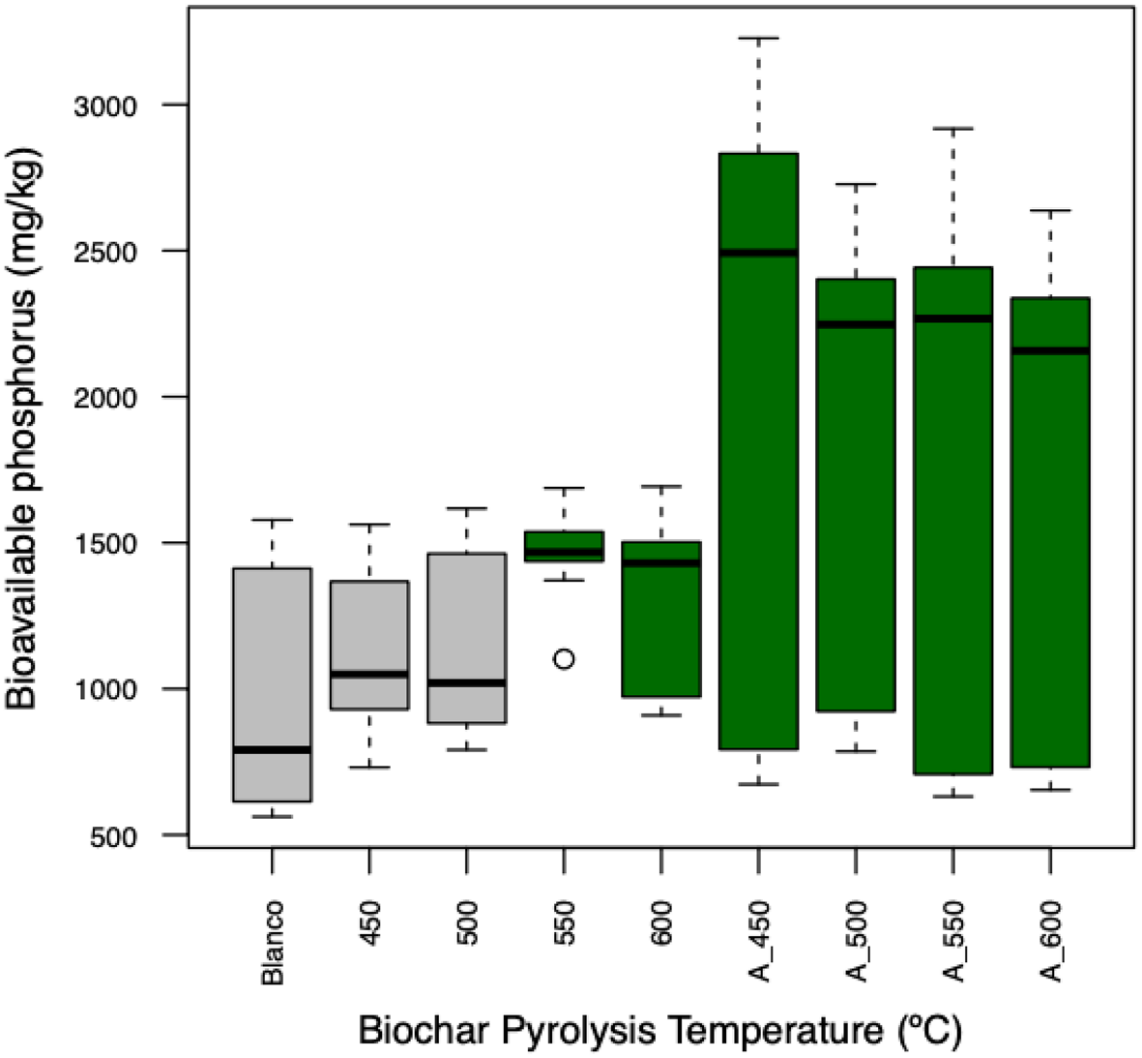
Concentration of soil bioavailable P. Green color indicates statistical significance differences compared to the control. Blanco (Control without biochar). 450, 500, 550, and 600 (P-loaded biochars produced using raw biochars). A_450, A_500, A_550, and A_600 (P-loaded biochars produced using activated biochars).

P-loaded biochars produced from raw biochars (550 and 600 °C) increased the soil bioavailable P concentration. The use of activated biochars to prepare P-loaded biochars increased bioavailable P regardless of the temperature (Fig. 1, Tables 4, 5). It is possible that for P-loaded biochars produced from raw biochars (550 and 600 °C) their effectiveness in improving bioavailable P was related to a higher content of basic cations (Ca, Mg, and Na) (Table 1). PO_4_^3−^ ions can form bridge bonds with the residual charge of ligand-bonded divalent cations, resulting in PO_4_^3−^ sorption onto biochars, thus increasing the bioavailability of P in the material and thus in the soil^12,31^. The increase in efficiency of all P-loaded biochars produced from activated biochars was also associated with the increase in surface area^32^. A higher specific area facilitates P from the KH_2_PO_4_ solution to be deposited in the pores, increasing the bioavailability of P in the material^12,33^. The capability of these materials to slowly release P keeps this element in bioavailable forms, preventing an excess of P in the soil solution, which can cause P to form insoluble precipitates with other soil elements^34^. This is explained by the diffusivity of P from the matrix to the soil solution and the desorption of P from the biochar when P is adsorbed through weak chemical bonds^12^. Overall, the use of P-loaded biochars produced from activated biochars could improve the bioavailability of P in tropical soils, which normally lack bioavailable concentrations of this element. From this point of view, the evaluated materials may be tempting for implementation.

### Soil Pb bioavailability

**Figure 2:**
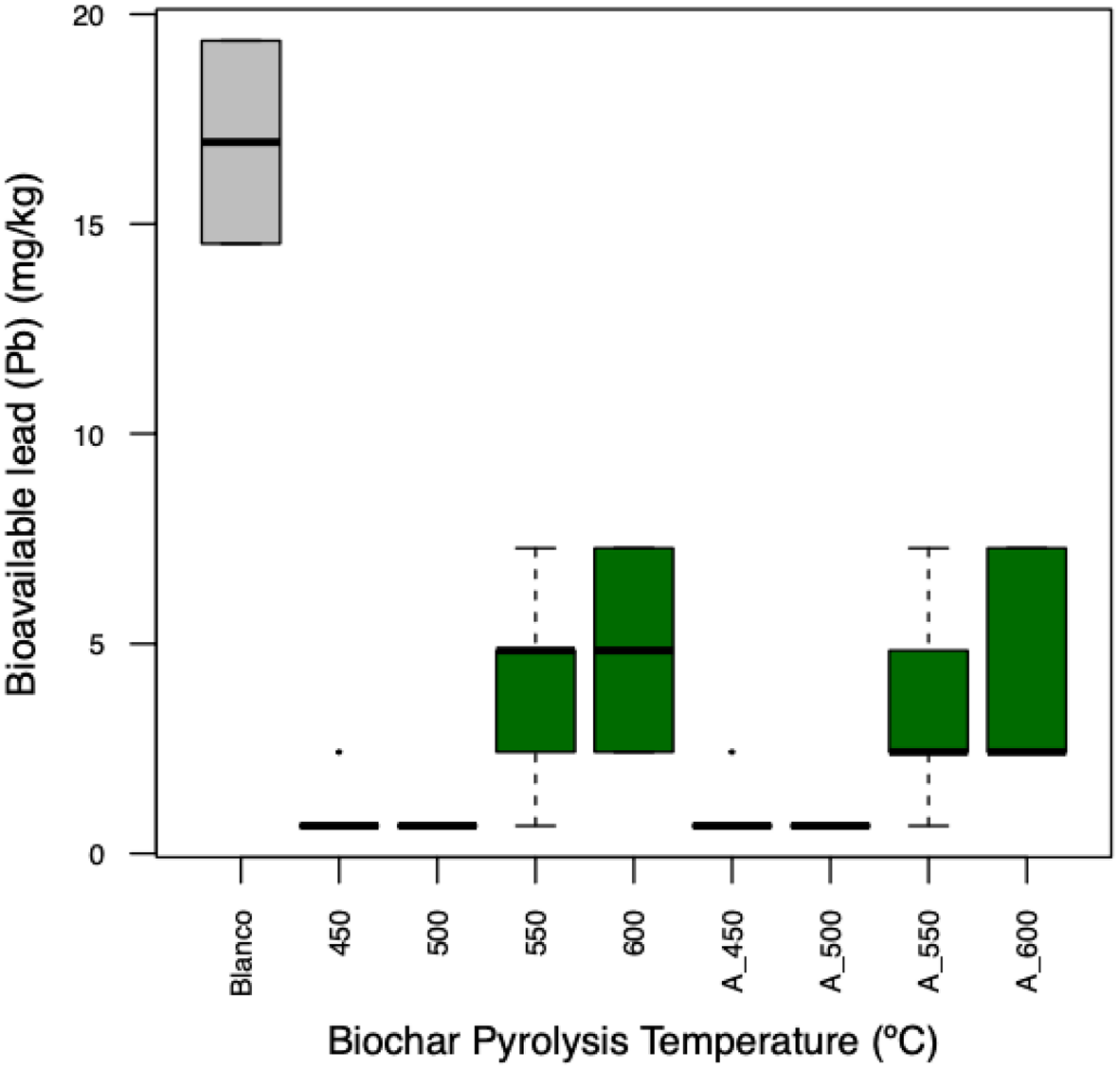
Concentration of soil bioavailable Pb. Green color indicates significant statistical differences compared to the control. Blanco (Control without biochar). 450, 500, 550, and 600 (P-loaded biochars produced using raw biochars). A_450, A_500, A_550, and A_600 (P-loaded biochars produced using activated biochars).

Incorporation of P-loaded biochars decreased the bioavailability of Pb in the soil, regardless of the production temperature and the use of raw or activated biochars (Fig. 2, Tables 4, 5). The biochars produced at pyrolysis temperatures between 401 and 600 °C have been effective in reducing the bioavailability of heavy metals such as Pb in soils^35^, and as has been shown in this study, the biochar produced at 500 °C is the one that presented the best results in bioavailability. This is also because the BET area is the highest (Table 3). It is possible that the remediation capacity of these materials is associated with ion exchange, complex formation, and/or co-precipitation^36^ or by indirect phenomena such as increased soil pH and ion exchange, as has been widely reported^35^. However, the materials obtained from biochar produced at 450 and 500 °C showed a greater decrease in bioavailable Pb (Fig. 2). Materials produced at these temperatures can have a greater diversity of surface functional groups associated with cation adsorption capability^9^. From an environmental point of view, the materials evaluated in this study could be effective for the remediation of Pb-contaminated soils. Possible mechanisms of action after P-biochar application should be analyzed.

**Table 3:** Chemical composition of the raw biochars determined by XRF.

Compound (wt%)	Biochar			
	450	500	550	600
SiO_2_	89.57	89.45	89.07	89.65
Al_2_O_3_	0.85	0.92	0.99	0.83
Fe_2_O_3_	1.12	1.26	1.38	2.28
CaO	1.57	1.85	2.07	2.02
MgO	0.57	0.57	0.63	0.73
SO_3_	0.09	0.08	0.27	0.14
K_2_O	5.48	5.11	4.73	3.81
Na_2_O	0	0	0.12	0.22
Ti_2_O_3_	0.21	0.23	0.23	0.26
P_2_O_5_	0.39	0.39	0.36	0.51
Mn_2_O_3_	0.11	0.13	0.14	0.15

### Biomonitoring

**Figure 3:**
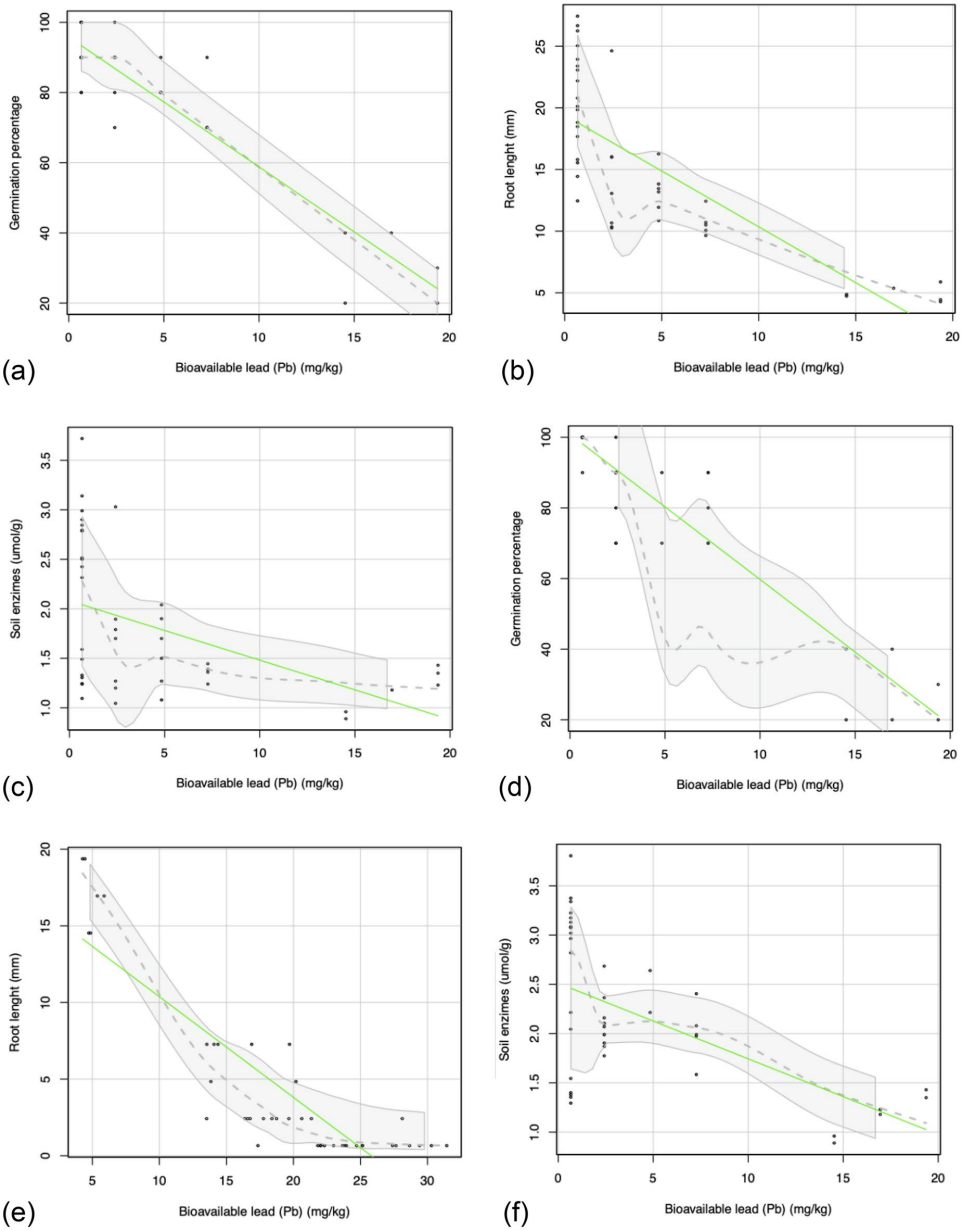
Relationship between soil Pb bioavailability and bioindicators (germination percentage, root length, and soil enzyme activity). P-loaded biochars produced using raw biochars (**a–c**). P-loaded biochars produced using activated biochars (**d–f**).

The bioindicators selected for biomonitoring in remediation were sensitive to changes in the bioavailable concentration of Pb (Fig. 3). For both the P-loaded biochars produced from the raw biochars and the activated biochars, it was observed that an increase in soil Pb bioavailability reduced the germination percentage and root length of radish (Fig. 3a,b,d,e). Also, urease activity decreased with increasing bioavailable Pb in the soil (Fig. 3c,f).

**Figure 4:**
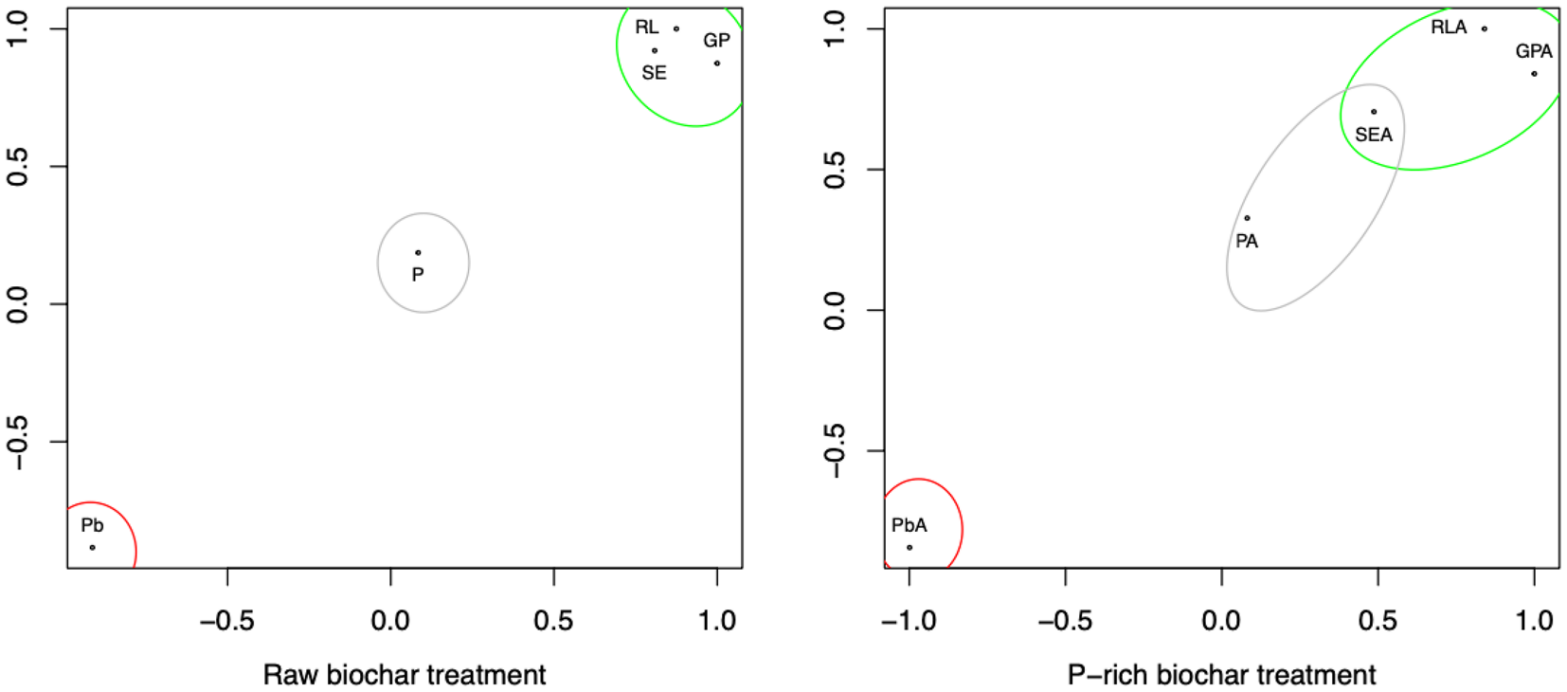
Principal component analysis.

Radish has been a model plant used in ecotoxicological studies^37^. Likewise, soil enzymes have been used as bioindicators in Pb-contaminated soils^38^. Principal component analysis (Fig. 4) shows that Pb bioavailability is opposite to germination and root elongation of radish and urease enzyme activity in soils. Bioavailable P was not related to increased germination or increased plant root length for P-loaded biochars produced from the original biochar (Fig. 4). In the case of P-loaded biochars produced from activated biochar, the increase in germination may have been favored by the increase in P bioavailability (Fig. 4). These results show that biochar activation not only remediates soil Pb but also improves radish root elongation and germination. The foregoing makes the application of P-loaded biochar to the soil improve its conditions, and remediation can be achieved in up to 15 days.

**Figure 5:**
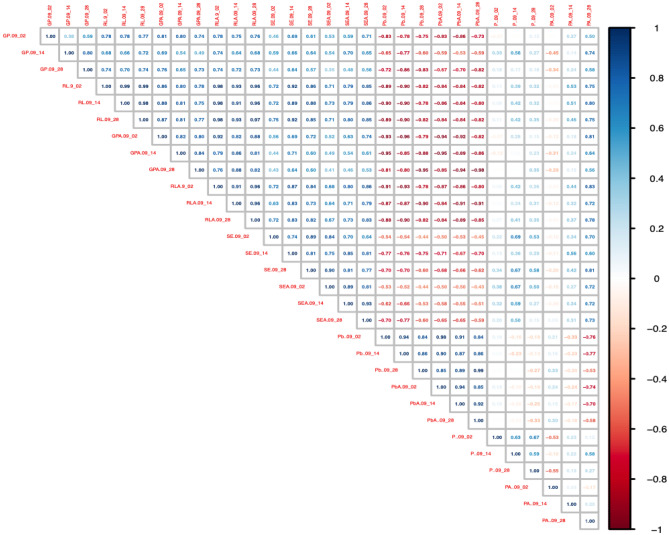
Correlation analysis.

Correlation analysis (Fig. 5) confirms that germination is a better indicator for the P-loaded biochars produced from the original biochar. Instead, root length and urease enzyme activity are good indicators for both P-loaded biochars produced from original biochar and activated biochar. From an economic and practical point of view, root length is a simpler bioindicator than the determination of enzyme activity. Biomonitoring becomes relevant in contaminated sites as it is an indicator of ecotoxicological risk^39–41^. Therefore, the use of a simple bioindicator is useful to infer how it reduces the risk in the soil associated with the decrease in the bioavailability of Pb. This study demonstrates that root length could be a simple bioindicator for biomonitoring the remediation of Pb-contaminated soils using P-loaded biochars. Likewise, the correlation analysis shows that the activation of biochar is essential not only to improve soil properties but also to reduce the bioavailability of Pb in the soil (Fig. 5). Another important factor found is that this material allows, as already mentioned, that in addition to decreasing bioavailability, it also increases the availability of P, leading to an increase in the germination of the applied bioindicator. Consequently, it can be inferred that in agricultural soils, this material could improve crops and reduce recovery times currently applied.

### Pb immobilization in P-loaded biochar

**Figure 6:**
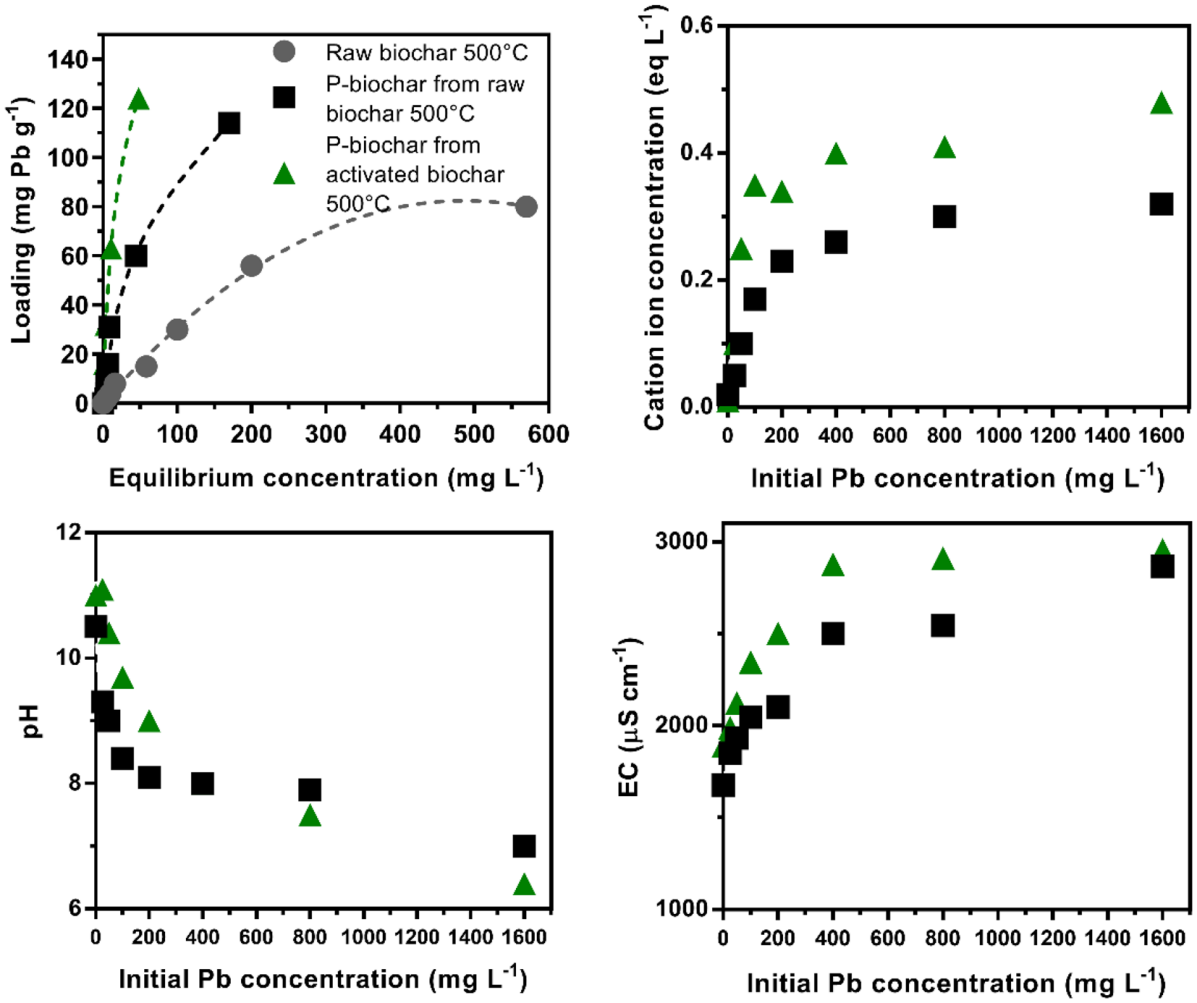
Excess adsorption of Pb from aqueous solution on Raw biochar (500 °C), P-biochar from raw biochar (500 °C), and P-biochar from activated biochar (500 °C). Dotted lines are guides to the eye. Cation ion concentration, pH, and EC of the filtrate as function of the Pb initial concentration.

Figure 6 shows the excess adsorption isotherms for the P-loaded biochar produced from the original biochar (500 °C), the P-loaded biochar produced from the activated biochar (500 °C) and the original biochar (500 °C). The two P-loaded biochars presented an L-type isotherm regardless of whether they were produced from the original biochar or from the activated biochar. The L-type isotherm indicates a high affinity of Pb for the adsorbent^9^. On the other hand, the biochar without loading with P showed an S-type isotherm, which indicates a high affinity of Pb with the material but only at high concentrations of Pb in the solution^9^. The results suggest that the loading process with P increases the adsorption of Pb by the biochar, even when Pb is found at low concentrations in the solution. This is ideal, since the concentrations of contaminants in the soil are generally low. From an environmental point of view, the best materials for remediation are those that adsorb the contaminant even at low concentrations, not only when it is in high concentrations^9,8^.

The L-type isotherm suggests that the adsorption of Pb in the biochar is due to chemisorption processes^9^. The results of the measurement of pH and EC in the post-adsorption filtrate show that as the adsorption of Pb in the material increases, the pH and EC of the solution also increase (Fig. 6). This suggests an ion exchange process, H^+^ is displaced by Pb^2+^, generating a decrease in pH. On the other hand, the increase in EC suggests the exchange of Pb^2+^ with basic cations^9^. The latter was verified by quantifying the Ca and Mg in the filtrate (Fig. 6). An increase in the concentrations of these two elements can be observed, indicating an ion exchange mechanism between Pb^2+^ with Ca^2+^ and Mg^2+^ in the material^9,42^. Ash content analysis showed an increase in ash in the two P-loaded biochars but not in the original non-P-loaded biochar (Fig. 6).

**Figure 7:**
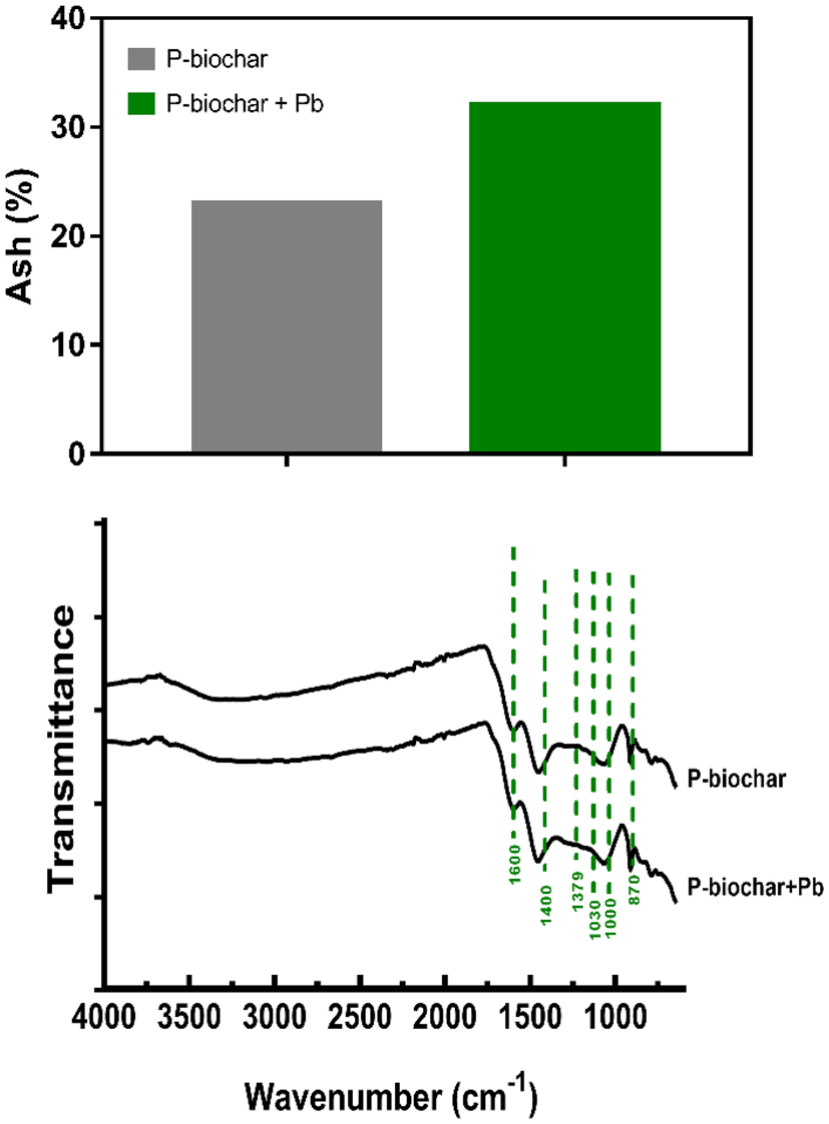
Ash content in P-biochar from activated biochar (500 °C) before (P-biochar) and after (P-biochar + Pb) Pb sorption. FTIR spectra of P-biochar from activated biochar (500 °C) before (P-biochar) and after (P-biochar + Pb) Pb sorption.

An increase in ash content in post-adsorption biochar has been associated with the formation of new crystalline phases in material^12^ (Fig. 7). It is possible that this increase in ashes could be due to the formation of Pb precipitates, such as PbHPO_4_, Pb_5_(PO_4_)_3_(OH), other^42^. This suggests that the co-precipitation of Pb with other elements on the biochar surface is a possible mechanism involved in Pb immobilization. The FTIR analysis is shown in Fig. 7. Changes in the intensity of functional groups PO_3_^4−^ (1000 cm^−1^) and CO_3_^2−^ were observed in the biochar post adsorption, which supports the possible formation of Pb precipitates^42^. Changes in the intensity of other functional groups (oxygen-containing functional group) are also observed, which suggests the formation of ligands is associated with Pb sorption^9^.

## Materials and methods

### Raw biochars

For this work, raw biochar has been produced from rice husk as described in previous works by the research group^28,29^. Table 3 presents the chemical composition of the biochar determined by X-ray fluorescence (XRF) (ZSX Primus Rigaku spectrometer, Tokyo, Japan). The biochar activation was carried out according to previous work^28,29^.

Table 3 shows the high content of silicon oxides in the coal, which is mainly due to the chemical composition of the biomass used. The low content of metals such as aluminum and iron are also evident, guaranteeing that they will not interfere with the cationic exchange process between phosphorus and lead. Likewise, elemental analysis of the biochar produced was carried out (Table 4), showing a high amount of C and low N and S content, which is also shown in Table 3.

**Table 4:** Physical–chemical characteristics of the biochar (ultimate analysis).

Parameter	Biochar				
	Rice husk	450	500	550	600
Nitrogen (wt% d.b.)	0.70	0.59	0.62	0.50	0.48
Carbon (wt% d.b.)	31.60	63.90	64.20	65.60	69.3
Sulfur (wt% d.b.)	0.01	0.28	0.31	0.20	0.27
Hydrogen (wt% d.b.)	4.35	1.52	1.25	1.30	1.28
Oxygen (wt% d.b.)	47.37	9.71	8.62	7.40	6.67

In the same way, the pore size has been analyzed (Table 5), for which a BET area analysis has been developed on the AutoChem II 2920 equipment (Micromeritics, Norcross, USA). All biochar samples present two types of pores: micropores and larger average pores. Micropores with a size of 2 nm. Among the four biochars produced, the one produced at 500 °C activated presents the most abundant pore volume at 2 nm, 0.354 cm^3^ g^−1^, which can allow a better exchange of phosphorus and lead in the soil^45^. It can be seen how all biochars when activated the surface area increases considerably.

**Table 5:** Specific surface area of the biochar studied.

Biochar		S_mb_ (m^2^ g^−1^)	Mean pore size (nm)	Micropore volume (cm^3^ g^−1^)
Activated	450	667.84 ± 0.18	2.583	0.325
Raw		65.98 ± 0.16	0.162	0.033
Activated	500	774.83 ± 0.34	2.645	0.354
Raw		128.32 ± 0.12	0.162	0.038
Activated	550	704.19 ± 0.04	2.723	0.345
Raw		106.74 ± 0.08	0.163	0.045
Activated	600	647.89 ± 0.45	2.837	0.214
Raw		100.28 ± 0.21	0.162	0.050

### P-loaded biochars

To produce P-loaded biochars, the raw biochars and activated biochars were immersed in a saturated KH_2_PO_4_ solution as described in Sepúlveda-Cadavid et al.^43^. Then, biochar was separated by filtration using a 0.45 µm membrane. Subsequently, it was washed with distilled water, dried at 60 °C and then stored. The bioavailable P content was determined with the Olsen method (Olsen-P)^12,44^. In brief, 1.0 g of solid with 20 ml of 0.5 M NaHCO_3_ solution (pH 8.5) was shaken (100 rpm) for 30 min, then the solid was removed by filtration (0.45 µm). The concentration of P was quantified using the molybdenum blue colorimetric method with a UV–Visible spectrophotometer at 800 nm (Thermo Scientific Model Genesys 10S UV–Vis).

### Soil sampling

Pb-contaminated soil was classified as Entisol as per USDA soil taxonomic classification. Soil samples were collected from the top layer (0–20 cm) using the methodology proposed by Muñoz-Romero et al.^45^, thus obtaining a composite soil sample. Afterward, the soil was air-dried, ground, sieved (< 2 mm), and stored. Soil properties were: pH (7.87), electrical conductivity (EC) (0.18 dS m^−1^), soil organic matter (SOM) (18.5%), cation exchange capacity (CEC) (10.5 cmol_(+)_ kg^−1^), Olsen-P (18.4 mg kg^−1^), sand (15%), silt (38%), clay (47%), and texture (clay loam soil).

### Soil incubation experiment

The experiments were carried out in a rhizobox, which has dimensions of 20 × 10 × 10 cm. The soil was arranged inside the rhizobox and then the percentage of biochar was added to each box at the top. This was mixed in the first centimeter of soil. The samples were left for 45 days, and bioavailable P and Pb measurements were made on days 15, 30, and 45, to evaluate their behavior over time. On the day of the sampling, these were taken from different parts of the rhizobox, and then they were mixed in an appropriate way. The samples were taken in duplicate.

### Soil Pb and P bioavailability

The 0.01 M CaCl_2_ solution was used to study Pb bioavailability. This method extracts water-soluble and readily exchangeable metals in soil samples^46^. For CaCl_2_–Pb extraction was use 1:5 soil to extractant ratio. Soil with a 0.01 M CaCl_2_ solution was shaken (100 rpm) for 2 h, then soil was removed by filtration (0.45 µm)^47^. The concentration of Pb in supernatants was determined by ICP-OES (ICP-OES Thermo Scientific ICAP6500 DUO kit Thermo Scientific equipment, Waltham, USA). The bioavailable P concentration was determined with the Olsen method^9,12^. For pH and EC measurements, a 1:2 soil:distilled water ratio was used^45^.

### Biomonitoring

For soil biomonitoring after the remediation experiment, two tests were selected: (i) phytotoxicity and (ii) enzyme soil activity. *Raphanus sativus* was selected as a response model^37^. The radish seeds were bought from the company Fercon, with a germination percentage of 90% and a purity of 99%. The toxicity of these soils, the germination rate (%) and radicle elongation (mm) of growing seeds were used^37,48,49^. A 1:2.5 soil: deionized water ratio was used to obtain soil–water extracts. At room temperature (25 °C), the samples were shaken for 2 h. The soil samples were then centrifuged at 4000 rpm for 15 min^50^. Soil–water extracts were used for phytotoxicity tests^37^. Soil–water extract (6 ml) was used to wet a filter paper (Whatman), which had been previously deposited in a 90 mm petri dish. Subsequently, 10 seeds were placed in each petri dish. The Petri dishes were placed in darkness for 24 h, then germination was measured. Subsequently, they were stored again in darkness for 24 h, and after that, the radicle elongation was measured. Control seeds were incubated only with deionized water. Three replicates were performed for each treatment. Urease activity was quantified based on the determination of the NH_4_ released when soil is incubated with a buffered urea solution^50^.

### Pb sorption

To theorize about the possible mechanisms involved in Pb immobilization in biochar, the biochar with the best performance in decreasing Pb bioavailability and increasing P bioavailability in soil, was selected for Pb adsorption tests from aqueous solutions. Thus, batch adsorption experiments were conducted to investigate the Pb immobilization of P-loaded biochar produced from activated biochar (500 °C) as a function of the Pb concentration in solution. To compare the effects of the activation and P-loading processes, the raw biochar produced at 500 °C and the P-loaded biochar produced with the raw biochar (500 °C) were included in this test. First, 0.5 g of material was mixed with 50 ml of 0.01 M KCl electrolyte solution and shaken for 24 h at room temperature (25 °C). Lead was added at concentrations of 0, 25, 50, 100, 200, 400, 800, and 1600 ppm using Pb(NO_3_)_2_. After, material was removed by filtration and Pb in the filtrate was quantified by ICP-OES. The excess adsorption isotherms were calculated according to Ref.^9^. To determine possible mechanisms related to Pb immobilization, pH, EC, Ca^2+^, and Mg^2+^ concentrations in the filtrate were determined. The concentration of Ca and Mg in the filtrate was quantified by ICP-OES. In addition, samples of the post-adsorption biochar (P-biochar from activated biochar 500 °C after and before Pb sorption) were used to perform FT-IR analysis and to determine the ash content. Superficial functional groups were determined by FT-IR using an infrared spectrometer (Thermo scientific Smart iTR Nicolet iS10), which was operating in a 4000–400 cm^−1^ spectral range with a resolution of 4 cm^−1^. Ash content was measured as the weight of the residue after samples were heated to 700 °C in an open crucible using a muffle furnace^12^.

### Data analysis

All data is presented as the mean values and standard deviation of three replicates. To conclude if the treatments done are effective, the Wilcox test was applied to determine if the results obtained are different from control in a statistically significant manner. Consequently, it is established that in the null hypothesis, the results are not statistically different, and the significance level sought is 5%. Therefore, if the p value found is less than the significance level, the null hypothesis is rejected. All statistical tests were performed using R software version 3.2.3 (https://cran-archive.r-project.org/bin/windows/base/old/3.2.3/).

### Plant ethical statement

This study complies with relevant institutional, national, and international guidelines and legislation.

## Conclusions

The addition of phosphorous-laden biochar to soil to improve its properties and mitigate lead contamination has been successful. It has been shown that with the application of 1% by weight of biochar to soils contaminated with lead, immobilization is achieved in a short time. This is because, according to the statistical results, time has not been an important factor. In the same way, it can be observed in the measurements made at 15, 30, and 45 days of the experiment, where it was possible to show that it is the first 15 days where this immobilization occurs. Biomonitoring with radish showed that when there is greater bioavailability of Pb in the soil, germination and root length are significantly reduced.

The best biochar found in this work is those produced at 500 and 550 °C. It can be said that biochar produced at 500 °C would be the most suitable because it is generated in greater quantity and reduces energy costs in production. This biochar improved soil remediation, retaining lead and releasing P so that the bioindicators used in this study had better results, such as greater germination and root length in the radish. However, the cost-energy production of a biochar at 550 °C is quite high, therefore, considering that both the 500 and 550 °C presented the same efficiencies and that producing a biochar at 500 °C is much cheaper, because this ends up being the best biochar.

Although for developing countries the price of biochar can be restrictive for its use, by improving the bioavailability of P, fertilization costs can be reduced, making this type of alternative more attractive to farmers. The use of biomasses with high P content could be evaluated to increase the concentration of this element in the biochar, reducing the amount of P to manufacture P-loaded biochar. Another option is the co-application of this material with other amendments rich in P, such as compost, this must be evaluated.

## Data Availability

All data generated during the current study are available from the corresponding author on reasonable request.
